# Nitric Acid in Whooping Cough

**Published:** 1862-05

**Authors:** S. W. Noble


### CHICAGO MEDICAL JOURNAL.

VOL. V.] MAY, 1862. [No. 5.

### ORIGINAL COMMUNICATIONS.

### NITRIC ACID IN HOOPING COUGH.

Read before the McLean County Medical Society, by

S. W. NOBLE, M. D.

Mr. President—By permission I would submit a short paper to the consideration of this Society, the reading of which will fully explain its object, and the universal prevalence of Hooping Cough in our County at present is sufficient apology for reading it at this time.

A few days ago while examining the able report of the Chairman of the Committee on Practical Medicine to the Illinois State Medical Society, I was astonished to find that he had made no mention of Nitric Acid in the treatment of Hooping Cough. He said that during the year this disease had prevailed as an epidemic in his own county. Finding nothing on the subject I was prompted to give my experience to the profession. I will not attempt to write an essay on Hooping Cough, or to present a review of the treatment that has been from time to time adopted. My object is to offer a plain relation of facts which I hope and believe will be of advantage to every member of the Society. And as my investigations all far from being thorough, and having but little authority for what I say on this matter, I would be generously indulged by the members present. Were it not the fact that you are all acquainted with me and know that I am one of the last men to be carried away with new cures, I would not make this report.

Dr. Goodbrake's description of the epidemic in De Witt County forcibly reminds me of one that visited our portion of McLean County in 1852 & '53. The epidemic raged for some time in a very malignant form. A large per-centage of the cases terminating fatally. This state of affairs became alarming to parents and physicians. We tried all the known remedies and some of the "domestic," but to little effect. I well remember my anxiety and professional responsibility was increased from the circumstance that I was at the time boarding with an excellent family of people, who had a couple of favorite children sick of Hooping Cough—one a daughter aged six years—the other a son two years younger, both of delicate constitutions and the son predisposed to Asthma and Croup. When they had been sick about six weeks, and were almost worn out by repeated spasms of coughing, unable to retain food upon their stomachs, their faces swollen from engorgement of the vessels, until their eyes were almost closed, frequent attacks of epistaxis, and all remedies usually prescribed in such cases tried to no purpose, including the Nitrate of Silver topically applied as recommended by Dr. Watson, I chanced to meet my uncle Dr. H. Noble, a member of this Society to whom I related my sad experience in the prevailing epidemic. He informed me that he had seen Nitric Acid recommended in some of the Journals as a cure for Hooping Cough and urged me to try it. But I was unable to see how the remedy, could cure the disease in question, and paid but little attention to what he said. I searched my library for the article he referred to but did not find it, and to this day have never read anything in the Journals on the subject—(since reading this paper I have found the article referred to on page

125, vol. 1, new series of this Journal.) Two or three days after the interview, the mother becoming more and more solicitous to have something more done for her children 1 related to her the conversation I had had with my uncle and proposed, if she was willing, to give the Acid a trial. I told her that it was an excellent tonic and could do no harm, but insisted upon her not expecting too much for the medicine and again informed her that Hooping Cough was a self-limited disease and must run its course. She was anxious to try anything that promised relief, for she was nearly exhausted from protracted watching and anxiety. So I put 1 ʒ Nitric Acid in a tumbler of sweetened water, and directed the oldest child to drink freely of that during the afternoon. The drink was palatable, and the mother being desirous to test the powers of the remedy induced the child to drink it all in about five hours. I then prepared some more which was given freely until bedtime, when the child went to sleep and rested all night without waking—the first good sleep she had had for a month. She never hooped again.

On the day following the boy took the same prescription with like effect. These children were so reduced with the croup that they had been "watched with" for weeks and every time they coughed during the night, and that was every 15 or 20 minutes, they had to be raised up and supported in the bed to prevent suffocation. The improvement in these cases was so sudden and well marked that we could not satisfactorily account for it in any other way than to attribute it to the Nitric Acid. A coincidence would not explain the change.

As before said Hooping Cough was common at the time, and I prescribed the Acid in nearly every case thereafter, and in uncomplicated cases with almost uniform success—I could give you case after case in which I have prescribed it; and if necessary I could procure genuine certificates that would startle a patent nostrum vender. But I deem such things uncalled for, and if I have said enough to induce the members present to give the article a trial, I will have accomplished my object in reading this paper, for if you once try it, I am certain that it will work as finely for you as it does for me. My formula for giving it is this: fill a glass-tumbler with sweetened water, and pour in nitric acid enough to make it as sour as good lemonade. This makes comparatively a pleasant drink, which I insist upon my patients *drinking freely*. I have no fears of his drinking too much, if it is sufficiently diluted. We have a delicacy in saying how much a patient may take. I have given 1 ʒ to a child six months old, within twenty-four hours. An adult male can take 1 ℥ per day, and plow right along.

The question will no doubt be asked, will it cut short the the disease? It will. How much?—This I will answer by asking, what is the usual course of the complaint? Of what number of days duration? Dr. Watson, who I believe is admitted on all hands to be good authority, says: "The ordinary duration of the disease is from six weeks to three months." He further says: "It will in all probability run a certain course, and our business is to conduct it evenly and safely to the end of this course."

According to my experience with the acid treatment, if it is commenced early and continued regularly, the disease will terminate in between one and three weeks and a-half, and the patients will never hoop. But the most striking examples of the curative properties of the acid, are where the patient has been coughing three or four weeks with no complications. Breathing and circulation natural between paroxysms of coughing. In such cases I have no hesitation in promising a cure in at least forty-eight hours. I prescribe no remedy with more confidence than I do this in the spasmodic stage of hooping cough. I have used it now for over nine years, and my experience has not been confined to any particular epidemic or locality. * * * * * *

